# Association between atherogenic index of plasma and coronary artery calcification progression in Korean adults

**DOI:** 10.1186/s12944-020-01317-4

**Published:** 2020-07-02

**Authors:** Ji Sun Nam, Min Kyung Kim, Joo Young Nam, Kahui Park, Shinae Kang, Chul Woo Ahn, Jong Suk Park

**Affiliations:** 1 Division of Endocrinology, Department of Internal Medicine, Yonsei University College of Medicine, Seoul, South Korea; 2 Severance Institute for Vascular and Metabolic Research, Yonsei University College of Medicine, Seoul, South Korea; 3 Division of Endocrinology, Department of Internal Medicine, Hallym University Kangdong Sacred Heart Hospital, Seoul, Republic of Korea; 4 Division of Endocrinology and Metabolism, Department of Internal Medicine, National Health Insurance Service Ilsan Hospital, Goyang, South Korea

**Keywords:** Atherogenic index of plasma, Coronary artery calcification progression, Coronary artery calcification score, Atherosclerosis, Cardiac computed tomography, Cardiovascular risk factor

## Abstract

**Background:**

Dyslipidemia is a well-known risk factor for cardiovascular disease (CVD). Recently, atherogenic index of plasma (AIP) has been proposed as a novel predictive marker for CVD, and few cross sectional studies have demonstrated a relationship between AIP and coronary artery disease. The present study investigated the association between AIP and the progression of coronary artery calcification (CAC) in Korean adults without CVD.

**Methods:**

A total of 1124 participants who had undergone CAC measurement at least twice by multi-detector computed tomography (CT) at a health check-up center were enrolled. Their anthropometric measurements and various cardiovascular risk factors were assessed. AIP was defined as the base 10 logarithm of the ratio of the concentration of triglyceride (TG) to high-density lipoprotein-cholesterol (HDL-C). CAC progression was defined as either incident CAC in a CAC-free population at baseline, or an increase of ≥2.5 units between the square roots of the baseline and follow-up coronary artery calcium scores (CACS) in subjects with detectable CAC at baseline.

**Results:**

CAC progression was observed in 290 subjects (25.8%) during the mean follow-up of 4.2 years. All subjects were stratified into three groups according to AIP. There were significant differences in cardiovascular parameters among groups at baseline. The follow-up CAC and the incidence of CAC progression increased gradually with rising AIP tertiles. In logistic regression analysis, the odds ratio for CAC progression was 2.27 when comparing the highest to the lowest tertile of AIP (95% CI: 1.61–3.19; *P* for trend < 0.01). However, this association was attenuated after adjustment for multiple risk factors (*P* for trend = 0.67).

**Conclusions:**

There is a significant correlation between AIP and the progression of CAC in subjects without CVD. Although AIP was not an independent predictor of CAC progression, AIP should be considered when estimating the current as well as future CVD risk, along with other traditional risk factors.

## Background

Cardiovascular disease (CVD) is the leading cause of morbidity and mortality worldwide. Coronary artery calcification (CAC), as determined by multi-detector computed tomography (CT), is a sensitive measure to detect the existence of early coronary atherosclerosis. Moreover, CAC is considered an important risk factor for cardiovascular events [1–3]. Dyslipidemia is one of the most important factors that contribute to CVD. The association between CVD and the traditional lipid measures, including total cholesterol (TC), low-density lipoprotein cholesterol (LDL-C), triglyceride (TG), high-density lipoprotein cholesterol (HDL-C), and Lipoprotein (a), has been well-demonstrated [4].

Recently, the atherogenic index of plasma (AIP), a logarithmically transformed ratio of molar concentrations of TG to HDL-C, has been suggested as a novel marker for atherosclerosis and CVD [5–7]. Elevated TG and a low HDL level are strong markers of cardiovascular diseases, and an increase of TG levels causes an increase in the small dense LDL level and ultimately increases CV risk [8]. Some studies have reported its superiority in predicting atherosclerosis compared to traditional lipid parameters [7, 8]. For example, in a large cohort study consisting of postmenopausal women undergoing coronary angiography, Guo et al. demonstrated AIP to be superior to traditional lipid indices for predicting coronary artery disease in univariate as well as multivariate regression analysis [9].

Most of the previous studies compared the AIP between patients with overt coronary artery disease (CAD) and controls, and showed inconsistent results, while some prior studies assessed the correlations between AIP and traditional CVD risk factors, including Framingham risk score [10–12]. To date, no existing study has investigated the relationship between AIP and early coronary atherosclerosis in relatively low-risk subjects without CVD. Furthermore, while CAC progression has been suggested to be a stronger predictor of CVD mortality compared to baseline coronary artery calcification score (CACS) or traditional cardiovascular risk factors [13], there is a lack of data on the relationship between AIP and CAC progression**.** Therefore, the present study aimed to investigate the relationship between AIP and CACS as well as CAC progression in Korean adults without CVD.

## Methods

### Study population

**Fig. 1:**
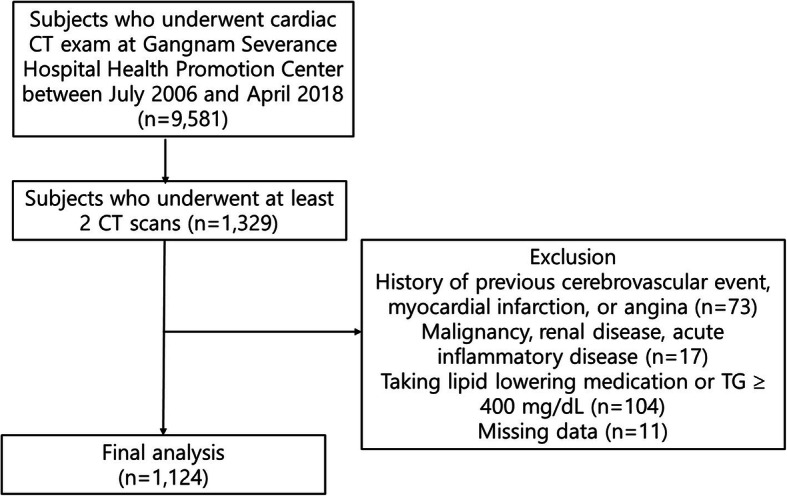
Flow chart of the study subjects

The present study was a retrospective longitudinal study. The study subjects comprised of 9581 Korean adults who underwent cardiac CT examination at Gangnam Severance Hospital Health Promotion Center in Seoul, Korea between July 2006 and April 2018. Initially, 1329 individuals who had undergone at least two cardiac CT scans were enrolled. Then, subjects with any malignancy, renal disease, acute inflammatory disease, missing data or a history of previous cerebrovascular event, myocardial infarction, or angina were excluded. Patients taking lipid-lowering medication were also excluded. Finally, 1124 subjects were analyzed (Fig. 1). This study was approved by the Institutional Review Board of Yonsei University College of Medicine (IRB approval number: 3–2019-0190).

### Anthropometric measurement and laboratory assessment

Subjects were examined after 12 h of fasting. They wore light clothing without shoes during body weight measurements. Body mass index (BMI) was determined using the following formula: body weight (in kg) divided by the square of person’s height (in meters). Measurements of systolic blood pressure (SBP) and diastolic blood pressure (DBP) were taken by trained nurses using an automatic blood pressure monitor (HEM-7080IC; Omron Healthcare, Lake Forest, IL, USA).

Blood sampling was performed for biochemical assessments including triglyceride (TG), total cholesterol (TC), high-density lipoprotein cholesterol (HDL-C), and fasting plasma glucose (FPG) using Hitachi 7600–120 automated chemistry analyzer (Hitachi, Tokyo, Japan). The calculation of low-density lipoprotein cholesterol (LDL-C) was done using the Friedewald formula. AIP was defined as the base 10 logarithm of the ratio of the concentration of TG to HDL-C, and each concentration was expressed in mmol/L [7].

Data on the subjects’ lifestyle habits, personal medical information, and medication history were collected through a questionnaire. A subject was considered to be a current smoker, if he/she smoked regularly in the last 6 months. A subject who consumed alcoholic beverages more than three times a week was considered to be a current drinker. Exercise with a moderate intensity for more than half an hour, at least three times a week, was defined as regular exercise. A subject was considered diabetic based on his/her previous history of diabetes, current use of antidiabetic medications, or the American Diabetes Association diagnostic criteria. SBP or DBP of greater than or equal to 140/90 mmHg and/or antihypertensive medication usage were considered as the criteria for hypertension.

### CAC assessment

CAC measurement was performed with a multi-detector CT scanner (Phillips Brilliance 64; Philips Medical System, Best, The Netherlands) using a prospective electrocardiogram-gating protocol with a step-and-shoot technique [14]. All subjects were in the supine position, and held their breath during the imaging process. One of the three trained radiologists, who were all blinded to the laboratory and clinical information, performed the analysis of coronary CT images. CACS was quantified automatically with dedicated software, and the severity was assessed using the Agatston score (Aquarius Workstation, TeraRecon, Inc., San Mateo, CA). A CACS above 0 was defined as coronary artery calcification. CAC progression was defined as either (A) incident CAC, indicating a baseline Agatston score of 0 but detectable CAC at follow-up examination in a population free from CAC at baseline [15], or (B) an increase of ≥2.5 units between baseline and final square root of CACS in participants with detectable CAC at baseline [16].

### Statistical analysis

Continuous variables are shown as the mean ± SD., whereas continuous variables with skewed distributions were expressed as the median with interquartile range. Chi square tests were performed to compare categorical variables, expressed as percentages. Analysis of variance was used for between-group analyses. The association between CAC progression and AIP was assessed by multiple logistic regression, after adjustment for any potential confounders. In the multivariate model, the following covariates were chosen due to their clinical importance and statistical significance in the univariate analysis: age, sex, BMI, SBP, LDL-C, exercise, alcohol, smoking, presence of diabetes or hypertension, and baseline Ln (CACS+ 1). Statistical analyses were performed using SPSS 25.0 (SPSS, Inc., Chicago, IL, USA), and *P* < 0.05 was considered statistically significant.

## Results

### Baseline characteristics

A total 1124 subjects were analyzed in this study. Table 1 shows the clinical and biochemical characteristics of the study participants. The subjects were stratified into three groups of T1, T2, and T3, with T1 being the lowest baseline AIP tertile and T3 being the highest tertile. Significant differences were observed in metabolic parameters among groups. SBP, DBP, BMI, as well as serum FPG, TC, TG, and LDL-C levels increased and HDL-C level decreased in the order of increasing AIP tertile. In addition, the highest AIP group had the greatest number of subjects with hypertension, diabetes, and current smoking habits. Alcohol intake and exercise habits were not significantly different between groups. Baseline CACS gradually increased with the increasing order of AIP tertile.

**Table 1:** Baseline characteristics of participants according to AIP tertiles

	T1	T2	T3	*P* value
N	376	373	375	
Age (years)	51.4± 8.0	52.0± 7.4	51.3±7.7	0.43
Sex (M/F)	182/194	281/92	331/44	
SBP (mmHg)	120.5±16.6	123.4±15.6	126.6±14.5	<0.01
DBP (mmHg)	74.8±10.5	77.6±9.6	80.1±8.7	<0.01
BMI (kg/m^2^)	22.9±2.8	24.1±2.7	25.4±2.8	<0.01
FPG (mmol/L)	5.08±0.80	5.40±0.80	5.67±1.11	<0.01
TC (mmol/L)	5.00±0.83	5.09±0.92	5.25±0.99	<0.01
TG (mmol/L)	0.75±0.18	1.21±0.26	2.17±0.67	<0.01
HDL-C (mmol/L)	1.60±0.29	1.28±0.22	1.06±0.2	<0.01
LDL-C (mmol/L)	3.04±0.76	3.3±0.82	3.37±0.89	<0.01
AIP	-0.31(-0.41, -0.24)	-0.03(-0.09, 0.04)	0.27(0.18, 0.39)	<0.01
HTN (%)	68(18.1)	102(27.3)	114(30.5)	<0.01
DM (%)	17(4.5)	27(7.2)	37(12.6)	<0.01
Alcohol (%)	46(12.2)	62(16.6)	64(17.1)	0.12
Smoking (%)	19(5.1)	44(11.8)	59(15.8)	<0.01
Exercise (%)	63(16.8)	73(19.6)	52(13.9)	0.12
Baseline CACS	13.3±46.9	23.8±79.1	25.8±90.3	<0.05
Categorical CACS				<0.01
0	289(76.9)	265 (71.0)	260 (69.3)	
0< and ≤10	23 (6.1)	29 (7.8)	26 (6.9)	
>10	64(17.0)	79(21.2)	89 (23.8)	
Baseline Ln (CACS+1)	0.75±1.53	1.02±1.78	1.03±1.77	0.03
CAC > 0 (%)	87 (23.1)	108 (29.0)	117 (31.2)	<0.05

Data are mean ± SD, number (percentage), or median (interquartile range)

Statistical significances were tested by Oneway analysis of variances among groups

*SBP* systolic blood pressure, *DBP* diastolic blood pressure, *FPG* fasting plasma glucose, *TC* total cholesterol, *TG* triglyceride, *AIP* atherogenic index of plasma, *HTN* hypertension, *DM* diabetes mellitus, *Alcohol* moderate drinking, *Smoking* current smoker, *Exercise* regular exercise of moderate intensity, *CACS* coronary artery calcium score

### Follow-up CACS

Table 2 shows the follow-up CACS and related parameters according to baseline AIP. The average follow-up period was 4.2 ± 2.2 years, and it was not significantly different among groups. Follow-up CACS and the incidence of CAC progression significantly increased in the order of increasing AIP tertile.

**Table 2:** Follow-up CAC-related parameters according to baseline AIP tertiles

	T1	T2	T3	*P* value
N	376	373	375	
Follow-up CACS	27.8±83.5	53.3±162.7	64.0±191.6	<0.01
Categorical CACS				<0.01
0	271 (72.1)	223 (59.8)	208 (55.5)	
<0 and≤10	14 (3.7)	27 (7.2)	29 (7.7)	
>10	91 (24.2)	123 (33.0)	138 (36.8)	
Follow-up Ln (CACS+1)	1.07±1.88	1.54±2.12	1.77±2.19	<0.01
Observation time (years)	4.1±2.2	4.2±2.3	4.3±2.2	0.32
CAC progression (%)	66 (17.6)	102 (27.3)	122 (32.5)	<0.01

Data are mean ± SD, number (percentage)

Statistical significances were tested by Oneway analysis of variances among groups

*CACS* coronary artery calcium score, *AIP* atherogenic index of plasma

**Fig. 2:**
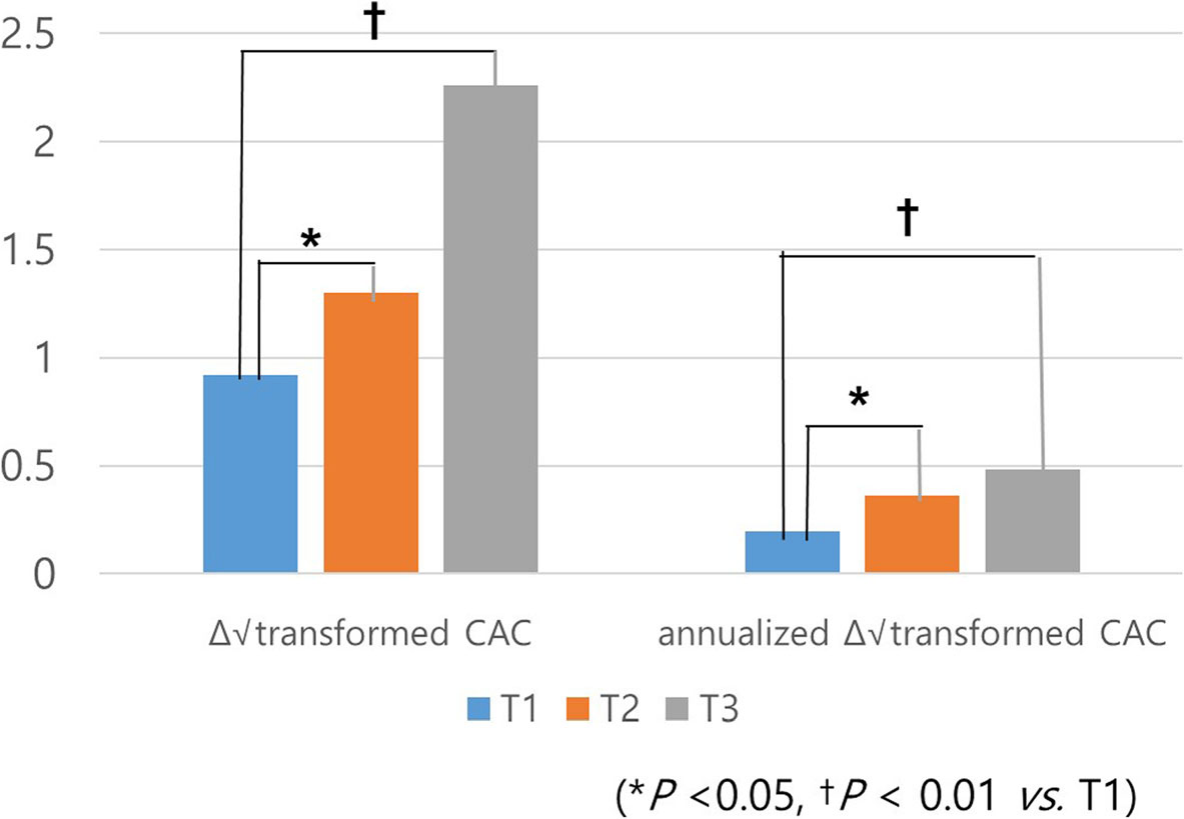
The change in coronary artery calcification according to AIP tertile Δ √transformed CACS and annualized Δ √transformed CACS values increased across the tertiles of AIP at baseline (T1: lowest AIP tertile, T2: second AIP tertile, T3: highest AIP tertile)

Figure 2 demonstrates that both the Δ √transformed CACS (T1, 0.90 ± 2.40; T2, 1.47 ± 3.42; T3, 2.01 ± 3.61; *P* < 0.01) and annualized Δ √transformed CACS (T1, 0.20 ± 0.70; T2, 0.36 ± 1.31; T3, 0.45 ± 0.81; *P* < 0.01) values increased across the tertiles of AIP at baseline. The group with the higher baseline AIP had the greater Δ √transformed CACS and also the annualized Δ √transformed CACS values.

### Association between CAC progression and AIP

The relationship between AIP and the progression of CAC was explored by categorizing the baseline AIP into tertiles, using the first tertile as the reference (Table 3, Supplementary Table 1). An unadjusted multiple logistic regression analysis revealed that, with T1 as the reference, the AIP levels for T2 and T3 increased the ORs for CAC progression. This relationship remained statistically significant after adjustment for sex and age. However, this association was attenuated after additional adjustments for BMI, SBP, FPG, LDL-C, exercise, alcohol, smoking, presence of diabetes and hypertension, and baseline Ln (CACS+ 1).

**Table 3:** Odds ratios and 95% confidence intervals for CAC progression according to AIP tertiles

	OR (95% CI)			*P* for trend
	T1	T2	T3	
AIP				
Model 1	1.00	1.77 (1.25-2.51)	2.27 (1.61-3.19)	<0.01
Model 2	1.00	1.37 (0.94-1.99)	1.65 (1.14-2.39)	0.03
Model 3	1.00	1.13 (0.75-1.70)	1.21 (0.80-1.85)	0.67

Model 1: Unadjusted

Model 2: Adjusted for age and sex

Model 3: Model 2 + BMI, SBP, FPG, LDL-C, exercise, alcohol, smoking, presence of diabetes and hypertension, and baseline Ln (CACS+1)

*BMI* body mass index, *SBP* systolic blood pressure, *DBP* diastolic blood pressure, *FPG* fasting plasma glucose, *LDL-C* low density lipoprotein cholesterol, *CACS* coronary artery calcium score

## Discussion

The present study showed a significant relationship between AIP and CAC, as well as the progression of CAC, over a 4-year period in Korean adults without CVD. These findings were consistent with previous studies that showed strong associations between AIP and cardiovascular risk factors and CVD. Furthermore, to the best of our knowledge, this study was the first to reveal a longitudinal association between AIP and CAC progression.

When the study subjects were categorized into tertiles according to AIP, those at the highest tertile had the highest BP, BMI, CACS, and adverse lipid profiles. Also, the incidence of diabetes, hypertension, alcohol drinking, and smoking were the highest in this group. In line with the results of the present study, prior studies demonstrated that AIP is an independent predictor of CAD among Chinese subjects, Chinese postmenopausal women, and very young adults [10, 11, 17]. Another recent study revealed that AIP predicts a plaque burden in intermediate CVD risk patients presenting with chest pain [18]. In addition, AIP was associated with various metabolic disorders, including fatty liver disease, hypertension, diabetes, and diabetic complications [19, 20]. While most of these studies were conducted on subjects with overt CAD or chest pain, or those diagnosed with diabetes, which is considered equivalent to CAD, this study excluded subjects with CAD or cerebrovascular disease, as well those undergoing lipid-lowering therapy. Despite the relatively low cardiovascular risk of the study population, higher AIP was still associated with higher CACS.

Furthermore, higher AIP was associated with the progression of CAC. While 17.6% of the subjects in the lowest AIP tertile showed CAC progression, 32.5% of the subjects in the highest tertile showed CAC progression. Moreover, the Δ √transformed CACS and the annual Δ √transformed CACS increased gradually across the tertile, indicating that the baseline AIP predicts the progression of CAD and possibly future coronary events.

Similar results were obtained when subjects were divided into low risk and high risk CVD groups according to AIP values. Those with AIP value of under 0.11 belonged in low risk group and those with AIP values higher than 0.21 in high risk group. There was a significant difference in follow-up CACS between low risk and high risk groups (40.6 ± 129.4 vs. 73.3 ± 220.2, *P* = 0.026), and high risk group showed a higher percentage or CAC progression compared to low risk group (22.6 vs. 32.9%, *P* = 0.03).

However, after adjusting for various conventional cardiovascular risk factors, such as blood pressure, glucose, LDL-C, exercise, alcohol, smoking, BMI, and the presence of hypertension and diabetes, the predictive value of AIP on CAC progression lost its significance. There are several possible reasons for this, including a near normal, narrow range of lipid parameters of subjects in the current study. Even the subjects in the highest tertile AIP had the mean TG level of 2.17 mmol/L and mean HDL-C level of 1.06 mmol/L. Previous studies showed that high TG and low HDL-C were closely associated with CAC, even more than LDL-C [21, 22]. If the range of AIP in this study was wider with more extreme values, it may have resulted in a significant relationship. Also, while AIP is an independent risk factor for CAD in a cross-sectional setting [10, 11], other factors may be more critical to the progression of CAD. For example, insulin resistance is an important risk factor for atherosclerosis [23], and our previous study also demonstrated that triglyceride-glucose (TyG) index, which is a surrogate marker of insulin resistance, is an independent predictor of CAC progression [24]. Although AIP has been reported to be related with insulin resistance in Type 2 diabetes patients, its relationship has been somewhat inconsistent [25, 26]. In addition, some genetic risk scores based one known GWAS SNPs associated with CVD and CVD risk factors have been studied, and genetic determinants were shown to influence the progression of CAC [27]. Also, although it was not assessed in this study, calcium regulatory mechanisms that affect bone formation and growth are also known to influence CAC [28].

Although AIP did not predict the progression of CAC, it does not imply that AIP is not a good predictor of CVD. Recently, there has been a controversy over the prognostic value of the repeated measure of CAC in predicting CVD [29]. While prior studies suggested the additive contribution of changes in CAC in the prediction of CV, other studies showed that CAC change was only the fifth strongest risk marker for CHD, following baseline CAC, gender, SBP, and total cholesterol [30]. Also, MESA demonstrated that a CAC change of greater than > 100 U/y was associated with coronary heart disease, independent of risk factors and baseline CAC score [30]. In other words, although AIP was not able to independently predict the progression of AIP, it does not mean that is a good predictive marker of future CVD, and that it may have a synergistic role with the baseline CACS.

### Study strengths and limitation

The strength of the current study lies on the fact that it was the first study to investigate a longitudinal association between AIP and CAC progression. While most of the previous studies were conducted on subjects with CVD, our participants were with a relatively low CV risk, without prior CVD history. Meanwhile, there are several limitations. First, since this was a retrospective, longitudinal study, not all of the potential confounding factors were controlled. For example, although subjects taking lipid lowering drugs were excluded, those on antiplatelet agents and anti-diabetic, anti-hypertensive drugs that could affect the progression of atherosclerosis and AIP were included. Since the medication history was based on a questionnaire, the information on dose and class of these drugs were not available, and therefore these effects were not considered in the analysis. Also, diet, exercise, smoking, and alcohol consumption patterns were not controlled or monitored during the follow-up period, which was variable. Second, the results of this study cannot be generalized. People with existing CAD as well as those with no CVD risk were unlikely to be included in the current study, which led to nearly normal plasma lipid levels and a narrow range of CACS. Moreover, this study only included subjects who voluntarily took repeated coronary CT scans for a health check-up, which could lead to a selection bias. In addition, the sex ratio of the study participants was skewed especially in AIP tertiles 2 and 3. However, a subgroup analysis on gender was not conducted due to a limited number of study subjects. Last, this study used the same definition of CAC progression as a previous paper [23], but there is no consensus on the optimal way to quantify CAC change.

## Conclusion

Recently, AIP has been suggested as a novel marker for atherosclerosis and CVD, and some studies have demonstrated its prognostic value to be superior compared to traditional lipid parameters. Despite aforementioned limitations, this study offers significant implications that are clinically relevant, as it is the first to investigate the association between AIP and CAC progression. In this study of Korean subjects without CAD, subjects with higher AIP had an increased risk for cardiovascular disease and higher CACS, and were more prone to CAC progression over a 4-year period. Although AIP was not an independent predictor of CAC progression, a well-controlled prospective study including subjects with and without CVD is warranted in the future to further confirm the prognostic value of AIP. Until then, AIP should be considered when estimating the current as well as future CVD risk, along with other traditional risk factors.

## Supplementary information

**Additional file 1: Supplementary Table 1.** Odds ratios for CAC progression according to AIP tertiles

## Data Availability

Not applicable.

## Abbreviations

CVD: Cardiovascular disease
CAC: Coronary artery calcification
CT: Computed tomography
TC: Total cholesterol
LDL-C: Low-density lipoprotein cholesterol
TG: Triglyceride
HDL-C: High-density lipoprotein cholesterol
AIP: Atherogenic index of plasma
CAD: Coronary artery disease
CACS: Coronary artery calcium scores
BMI: Body mass index
FPG: Fasting plasma glucose
SBP: Systolic blood pressure
DBP: Diastolic blood pressure
TyG: Triglyceride-glucose
