# Supplementary material for: Association between atherogenic index of plasma and coronary artery calcification progression in Korean adults
Source: Lipids Health Dis. 2020 Jul 2;19:157. doi: 10.1186/s12944-020-01317-4 (PMC7331149; doi:10.1186/s12944-020-01317-4)
Supplement: Supplementary file 1 — Additional file 1: Supplementary Table 1. Odds ratios for CAC progression according to AIP tertiles [file 12944_2020_1317_MOESM1_ESM.docx]

**Supplementary Table 1. Odds ratios for CAC progression according to AIP tertiles**

|  | | | | | | | | | | | | | | | | | |
| --- | --- | --- | --- | --- | --- | --- | --- | --- | --- | --- | --- | --- | --- | --- | --- | --- | --- |
|  | | | B | | | S.E. | Wald | | | DOF | *P*-value | | Exp(B) | | | Confidence Interval | |
|  |  |  |  |  |  |  |  |  |  |  |  |  |  |  |  | Lower | Upper |
| Model 1 | aip T1 | |  | | |  | 22.185 | | | 2 | .000 | |  | | |  |  |
|  | aip T2 | | .570 | | | .179 | 10.186 | | | 1 | .001 | | 1.768 | | | 1.246 | 2.508 |
|  | aip T3 | | .818 | | | .175 | 21.895 | | | 1 | .000 | | 2.265 | | | 1.608 | 3.190 |
|  | Constant | | -1.547 | | | .136 | 130.212 | | | 1 | .000 | | .213 | | |  |  |
| Model 2 | aip T1 | |  | | |  | 6.968 | | | 2 | .031 | |  | | |  |  |
|  | aip T2 | | .314 | | | .191 | 2.719 | | | 1 | .099 | | 1.369 | | | .942 | 1.989 |
|  | aip T3 | | .500 | | | .189 | 6.966 | | | 1 | .008 | | 1.649 | | | 1.137 | 2.391 |
|  | Sex | | -1.246 | | | .203 | 37.852 | | | 1 | .000 | | .288 | | | .193 | .428 |
|  | Age | | .068 | | | .010 | 50.539 | | | 1 | .000 | | 1.070 | | | 1.051 | 1.091 |
|  | Constant | | -4.639 | | | .536 | 74.998 | | | 1 | .000 | | .010 | | |  |  |
| Model 3 | | aip T1 | |  |  | | | .806 | 2 | | | .668 | |  |  | |  |
|  |  | aip T2 | | .125 | .207 | | | .362 | 1 | | | .547 | | 1.133 | .754 | | 1.701 |
|  |  | aip T3 | | .193 | .215 | | | .803 | 1 | | | .370 | | 1.213 | .795 | | 1.849 |
|  |  | Sex | | -1.078 | .228 | | | 22.412 | 1 | | | .000 | | .340 | .218 | | .532 |
|  |  | Age | | .043 | .011 | | | 14.548 | 1 | | | .000 | | 1.044 | 1.021 | | 1.067 |
|  |  | BMI | | .040 | .031 | | | 1.683 | 1 | | | .194 | | 1.041 | .980 | | 1.105 |
|  |  | SBP | | .003 | .007 | | | .207 | 1 | | | .649 | | 1.003 | .990 | | 1.016 |
|  |  | FBG | | .003 | .006 | | | .165 | 1 | | | .685 | | 1.003 | .990 | | 1.015 |
|  |  | LDL-C | | .008 | .002 | | | 10.212 | 1 | | | .001 | | 1.008 | 1.003 | | 1.013 |
|  |  | Alcohol | | .092 | .221 | | | .174 | 1 | | | .676 | | 1.097 | .711 | | 1.692 |
|  |  | Smoking | | -.223 | .255 | | | .763 | 1 | | | .382 | | .800 | .486 | | 1.319 |
|  |  | Exercise | | .014 | .207 | | | .004 | 1 | | | .947 | | 1.014 | .675 | | 1.522 |
|  |  | Diabetes | | .282 | .348 | | | .658 | 1 | | | .417 | | 1.326 | .671 | | 2.620 |
|  |  | HTN | | .180 | .218 | | | .677 | 1 | | | .411 | | 1.197 | .780 | | 1.836 |
|  |  | lncac | | .275 | .044 | | | 39.190 | 1 | | | .000 | | 1.317 | 1.208 | | 1.435 |
|  |  | constant | | -6.185 | 1.249 | | | 24.506 | 1 | | | .000 | | .002 |  | |  |
|  | | | | | | | | | | | | | | | | | |

Model 1: Unadjusted

Model 2: Adjusted for age and sex

Model 3: Model 2 + BMI, SBP, FPG, LDL-C, exercise, alcohol, smoking, presence of diabetes and hypertension, and baseline Ln(CACS+1)
